# Retention of Urine after Labor

**Published:** 1847-04

**Authors:** James R. Dow

**Affiliations:** Colchester, Ct.


### 14. *Retention of the Urine after Labor.*

[Communicated for the Boston Med. and Surg. Jour.]

*Dr. Bedford—Dear Sir:* The case detailed below was intended solely for your inspection, but as I find it possessed of more than an ordinary share of interest, I am induced to offer it for publication, believing that it will serve to remind my junior brethren of *the necessity in all cases of* "*tracing effect to its proper cause*," and that it will also admonish my seniors in the profession, that *they* too are fallible and liable to err.

June 28th, 1845, I was requested to visit Mrs. Samuel Mitchell, in an adjoining town. On my arrived at the house, I received the following history from her physician, Dr. H.* Mrs. M. had given birth to a child ten days previous, the labor not being unusually long or severe. She appeared to be "doing well," until the morning of the 27th, when she remarked to her nurse that the room was becoming dark, and immediately she was seized with convulsions. From these, she would, at first partially recover, but was soon seized with another more severe than that which had preceded it; having had no less than nine distinct and well marked convulsions on that day. A messenger was despatched for Dr. H., soon after Mrs. M. was attacked, but he being absent, a neighboring physician, Dr. T., was called. He thought her to be suffering from puerperal convulsions, and immediately resorted to venesection. Dr. H. saw her in the evening, agreed with Dr. T. as to the diagnosis, practised venesection, and commenced giving tinct. stramonium, but what else, has passed from my mind. Dr. T. visited her the next morning, and finding the convulsions had returned with increased severity, he applied a blister to the back of the neck, and one upon the inner side of each leg. He also directed an enema of starch and laudunum, and a continuance of the treatment as prescribed by Dr. H. the evening previous. The abdomen being greatly distended, they applied fomentations. As the patient was evidently growing worse, farther counsel was desired; hence the occasion for sending for me. With this account, I proceeded to examine the patient. I found her suffering from coma, being wholly unconscious of what was going on around her; pulse 85, but hard; no unnatural degree of heat about the head; the condition of the pupils I do not recollect. There was incontinence of urine, and had been for a day previous. Upon examining the abdomen, *I found the bladder enormously distended, its fundus reaching the umbilicus.* I at once suggested the use of the catheter, and at the request of Dr. H. introduced it, and *drew from the patient more than one gallon of urine.* Of course the distension

* Drs. T. and H. have had considerable experience in their profession; the first having been in business nearly twenty, and the latter ten years.

of the abdomen was quickly and completely removed. In order to guard against inflammation, I advised farther depletion, which was adopted. With my fingers upon the pulse, a vein was opened, and as soon as its hardness appeared to yield, the bleeding was stopped. A cathartic (one of calomel and jalap) was then given and after its operation, was followed with liberal doses of spts. nit. dulce. By the frequent introduction of the catheter, and by the contined use of the diuretic, alternating with the sup. tart. potass. in doses sufficient to operate as a cathartic, I had the satisfaction to hear that Mrs. M. had fully recovered.

There are some points in the history of the above case that are worth being remembered; and,

1st. The manner of attack; the patient being of a sudden seized with convulsions, while friends, nurse and physician supposed her convalescing.

2d, The *absence of all pain*, the patient having at no time given any intimation that she experienced the least uncomfortable sensation about the bladder; hence I incline to the opinion that the distension of the bladder commenced in the months of pregnancy. Chailly in his admirable treatise on midwifery, page 220, speaking of cases where there is a retention of urine, says, "Happy indeed if the error is soon discovered ; for women, through an inconceivable ignorance of their medical attendants, have been known to succumb, with the most excruciating sufferings, and all owing to extreme distension of the bladder." But in the above case the patient complained of no pain, made no complaint, anticipated that she would soon be restored to the pleasures of society, when, suddenly, vision is rendered imperfect, reason is dethroned, and convulsions and coma attack the patient with great severity; yet all this caused by a retention of urine.

3d. Convulsions preceding and for a while accompanying coma. That the coma was caused by a partial suppression of the urine, the last produced by its retention, I think there can be no doubt. But what gave rise to the convulsions? Is it probable that there was a congestion of the brain? I think not. I am disposed to attribute it, like the coma, to the suppression of the urine; the blood thereby being rendered highly irritating, it is easy to see that as this was diffused throughout the brain and whole nervous system, disorder would be very likely to be induced.

I am not aware of any other case of retention or suppression of urine, that was followed by coma, where it was preceded by, and accompanied with, convulsions. If you have met with any similar instance in your own practice, or know of any upon record, please inform me.

In conclusion, permit me to say to you, likewise to your associates in the University, that I am not unmindful of the numerous favors I have received from your and their hands. I call to mind, with feelings of the highest pleasure, the many days I have spent in listening to valuable precepts you all labored so arduously to inculcate. Let me assure you that my alma mater is not forgotten; that in my intercourse with society, I feel that "my interests are her interests," and with a "watchful eye" shall sacredly guard its reputation. That its prosperity may ever continue, is the fervent prayer of

Your obedient servant,

*Colchester*, *Ct.*, *March* 29, 1847. JAMES R. DOW.

